# No improvement in long-term wear and revision rates with the second-generation Biomet cup (RingLoc) in young patients: 141 hips followed for median 12 years

**DOI:** 10.3109/17453674.2011.636672

**Published:** 2011-11-25

**Authors:** Bart Boesenach, Huub JL van der Heide, Rob GHH Nelissen

**Affiliations:** Department of Orthopaedic Surgery, Leiden University Medical Center, Leiden, the Netherlands

## Abstract

**Background:**

A number of excellent results with the cementless titanium femoral component of the Mallory Head Total Hip Replacement have been published. Unfortunately, these excellent results have been counteracted by the poor performance of the cementless titanium acetabular components. In 1994, the HexLoc acetabular component was replaced with a second-generation design, the RingLoc. We hypothesized that the new generation would have improved the results.

**Methods:**

We retrospectively studied 111 consecutive patients (150 hips) younger than 55 years. Median follow-up time was 14 (6–18) years for the HexLoc and 10 (1–14) years for the RingLoc. 7 patients were lost to follow-up and 7 patients died. The 10-year survival rate, radiographic liner wear, and radiographic signs of prosthesis failure were compared between the 2 acetabular components.

**Results:**

The Kaplan-Meier survival estimate with revision for any reason as the endpoint showed a 10-year survival of 89% (95% CI: 81–97) for the HexLoc and 92% (CI: 85–98) for the RingLoc. The mean annual wear rate for the HexLoc was 0.16 (SD 0.16) mm and it was 0.15 (0.1) mm for the RingLoc (p = 0.3). The radiographic signs of failure were equally distributed between the 2 groups.

**Interpretation:**

Compared to the HexLoc type, the RingLoc system did not improve the mean percentage survival at 10 years; nor did it reduce the liner wear. Despite correction of the known design flaws in the HexLoc design, the RingLoc system did not show a clinically relevant improvement compared to its predecessor.

Tapered designs for cementless femoral components have shown excellent clinical and radiographic results. Numerous authors have reported that these stem designs provide reliable fixation in the long term (Rothman et al. 1996, Hellman et al. 1999, Sakalkale et al. 1999, Mallory et al. 2001, Meding et al. 2004). In addition, these tapered cementless femoral designs have also been shown to give outstanding results in younger patients (Ellison et al. 2006).

Unfortunately, the results with cementless acetabular components do not match the success with their femoral counterparts (Bourne et al. 2001). High rates of wear have been reported, which can cause periprosthetic osteolysis (Harris 2004) and subsequent loosening of the component.

One of the components associated with inferior results is the cementless acetabular cup with a HexLoc liner (Biomet Inc., Warsaw, IN) locking system (Hozack et al. 1996, Puolakka et al. 1999). The HexLoc system was withdrawn from the market in 1994 and replaced by a new acetabular cup design with a RingLoc liner locking system (also Biomet).

We hypothesized that the new acetabular cup combined with the RingLoc liner locking system would have improved the 10-year survival in younger patients as compared to the former acetabular cup with the HexLoc liner locking system. Secondly, we hypothesized that the new cup would show less linear wear and would have reduced the number of radiographic failures.

## Patients and methods

### Patients (Table 1)

We conducted a retrospective cohort study on all consecutive cementless total hip prostheses (Mallory Head; Biomet Inc.) in patients younger than 55 years at the index operation, all inserted at the Leiden University Medical Center between 1990 and 2000. 111 consecutive patients (150 hips) were included. At final follow-up in 2008, 7 patients were lost to follow-up. 6 of them were living outside the Netherlands and 1 patient could not be traced. 7 patients (12 hips) died during the study period for causes unrelated to their hip surgery. 1 of these patients (1 hip) had been revised before death. All deceased patients were included in the survival analysis. This left 104 patients (141 hips) with a median follow-up of 12 (1–18) years for review. The patients were divided into 2 groups based on the acetabular component that was used, HexLoc (the H group) or RingLoc (the R group).

**Table 1.** Baseline characteristics of the 2 groups

	HexLoc	RingLoc
Total number of hips	64	77
Males/females	23/41	32/45
Mean age at surgery	41 (17–55)	42 (15–55)
Previous hip surgery	19	8
Mean preoperative HHS	43 (14–73)	43 (4–74)
Average BMI	24	24
Additional screw fixation	11	9
Deceased	5	6
Etiology		
Rheumatoid arthritis	23	24
Ankylosing spondylitis	10	9
Femoral head necrosis	9	16
Primary osteoartritis	7	9
Hip dysplasia/luxation	6	8
Trauma	1	3
Other	8	8

Median follow-up for the H group was 14 (6–18) years, and for the R group it was 10 (1–14) years. Previous resurfacing hip surgery had been performed in 19 hips in group H, including 17 Gerard double-cup arthroplasties (Duijsens et al. 2005) and 2 Thomine cups (a monopolar design). In group R, only 8 hips had had a previous operation, 4 Gerard double-cup arthroplasties, 3 Thomine fitted cups, and 1 Aufranc Smith-Peterson cup.

### Surgery

A direct lateral hip approach was used in all patients. In 13 cups in group H and in 9 cups in group R, screws were used for additional stability of the acetabular components. Additional screws were used based on the surgeon's perception of initial stability of the acetabular component during surgery. Indications for revision surgery were similar in both groups; in both groups revision was planned for end-stage liner wear or radiographic loosening of the acetabular component. None of the femoral stems had to be revised during the follow-up period.

### Implants

The Mallory Head porous coated femoral stem made of Ti6Al4V (Biomet) was used in all of the hips, combined with a 28-mm CoCr head in all cases but 2, where a ceramic head was used.

The Mallory Head (Biomet, Warsaw, IN) porous coated acetabular cup was used—combined with the HexLoc liner locking system in all cases—until it was withdrawn from the market in 1994. It was replaced with the RingLoc system, which was then used at our center. All cups had holes for screw fixation. The older cups combined with the HexLoc had 12 screw holes whereas the second generation has 7 holes for additional screw fixation of the cup with respect to the bone. The HexLoc system had a cylindrical shape at the inside of the acetabular metal shell, which caused the liner to be asymmetric and thinner at the edges (Hozack et al. 1996). The RingLoc acetabular cup does not have this liner incongruence at the edge. The size distribution of acetabular components was similar in both groups, the median being 52 (45–70) mm (Mann-Whitney U test; p = 0.2). The bearing materials used in both groups were ArCom liners made from 1900H ultra-high molecular weight polyethylene resin.

### Clinical evaluation

For evaluation, demographic data, previous surgery, radiographic variables, and clinical scores were collected from the medical records. All patients were assessed within 2 years of the study date to ensure recent data, either as part of their routine follow-up or at an extra follow-up for the current study. A prospective Harris hip score was taken preoperatively. At the last follow-up, a final Harris hip score was obtained for measurement of patient outcome.

### Radiographic evaluation

All radiographs were assessed by one observer who was blinded regarding all patient-related information. Anteroposterior (AP) non-weight bearing pelvic and femoral digital radiographs were taken at the last follow-up visit. These radiographs were compared to the radiographs taken immediately postoperatively. Radiographs were evaluated for signs of periprosthetic radiolucent lines, cortical hypertrophy, and bone resorption. The inclination of the acetabular cup was measured by drawing a horizontal line through the inferior edge of the teardrops and a second line through the superior and inferior edge of the acetabular cup (Wan et al. 2008). The angle between these 2 lines was measured.

Linear wear was measured as described by Dorr and Wan (1995) and Ebramzadeh et al. (2003). Periacetabular radiolucencies and osteolysis were assessed according to DeLee and Charnley (1976). The annual wear was calculated by dividing the total wear by the total follow-up time in years.

The cup was considered radiographically loose if a radiolucent line was wider than 2 mm and extended along all 3 zones of the acetabular component (Mann et al. 2002).

### Statistics

Survivorship analysis was done according to Kaplan and Meier. Survival estimates were deemed sufficiently accurate when at least 15 hips were still at risk (Dorey 2004). A log rank test was used to test for the difference in mean survival time between the 2 groups. To account for bilateral hips, we performed the survival analysis twice: including the 2 hips and randomly including 1 of the 2 hips. The difference in risk was calculated using the number of events (revisions) and persons at risk 10 years after the index operation.

Differences between variables with a normal distribution, including the annual wear rate, were tested using a linear mixed models analysis. This test is essentially a t-test, taking into account the bilateral hips.

All statistical analyses were performed using SPSS version 14.0. Statistical significance was assumed with p-values of < 0.05.

## Results

### Revisions

No femoral stems were revised, giving a mean 16-year survival of 100% with 16 hips at risk. During follow-up, 33 acetabular components were revised, either for liner wear or for acetabular loosening, or both. 25 revisions (22 liner exchanges, 3 cup revisions) were performed in the H group, as opposed to 8 (5 liner exchanges, 3 cup revisions) in the R group.

The subgroup analysis showed that at final follow-up, 28 of the 114 hips with no prior hip resurfacing were revised: 22 of them were liner revisions and 6 were cup revisions. In the resurfacing group, 6 of 27 hips had a liner revision and none of the metal-backed cups had to be revised.

### Survival analysis

The 10-year survivorship in group H was 89% (95% CI: 81–97) with 55 cases at risk, and it was 92% (CI: 85–98) with 41 cases at risk in group R (Table 2). The observed difference of 3% in 10 years was not statistically significant (p = 0.5). Also, there was no significant difference when we randomly excluded one of the bilateral hips (p = 0.2).

**Table 2.** Survival estimates for the 2 groups (95% CI)

Survival (years)	HexLoc	At risk	RingLoc	At risk
5	100%	63	100%	69
10	89% (81–89)	55	92% (85–99)	41
12	77% (67–87)	47	86% (77–96)	15
16	49% (34–63)	16		

**Figure 1.**
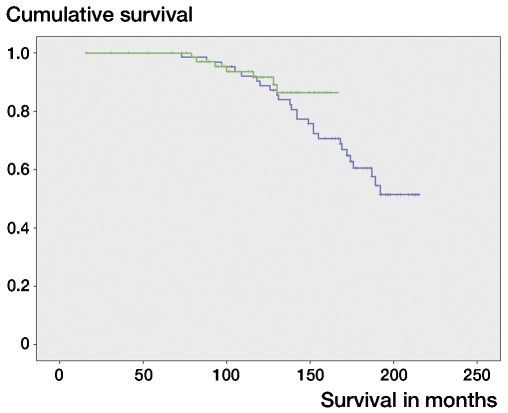
Kaplan-Meier survival curve. Blue: HexLoc; green: RingLoc.

The Kaplan-Meier survival curves for the 2 groups were similar up to 12 years of follow-up (Figure 1). After 12 years, an insufficient number in group B were at risk. The difference in risk between the 2 groups at 10 years was 0.005 (CI: –0.13 to 0.14). The subgroup analysis showed a slightly higher 10-year survival for the resurfacing group—91% (CI: 79–100) vs. 90% (CI: 84–96)—but the difference was not statistically significant (p = 0.4). The 10-year survival for patients with rheumatoid arthritis was higher—at 93% (CI: 86–100) vs. 88% (CI: 81–96)—but the difference was also not significant. (p = 0.3).

### Liner wear

Mean annual liner wear in group H was 0.16 (CI: 0.12–0.20; range: 0.00–1.13) mm/year. Group R showed a similar wear rate of 0.15 (CI: 0.13–0.17; range: –0.01 to 0.41) mm/year (p = 0.7).

A small effect of a previous resurfacing arthroplasty was seen on liner wear. In this subgroup, the mean liner wear was slightly higher at 0.18 (CI: 0.12–0.25) mm/year, as opposed to 0.15 (CI: 0.12–0.18) mm/year for those with no previous resurfacing, but this was not significant (p = 0.3).

A moderate effect of rheumatoid arthritis was seen on the annual wear. In this subgroup, it was slightly lower at 0.12 (CI: 0.07–0.17) mm/year, as opposed to 0.17 (CI: 0.14–0.21) mm/year in those with no rheumatoid arthritis, but this did not reach statistical significance (p = 0.08).

### Radiographic results

The mean postoperative cup inclination was 46 (30–72) degrees in the HexLoc group and 45 (26–61) degrees in the RingLoc group.

**Figure 2.**
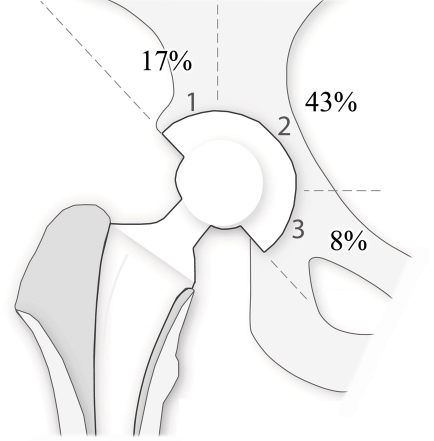
Radiolucencies around the acetabulum in the HexLoc group.

**Figure 3.**
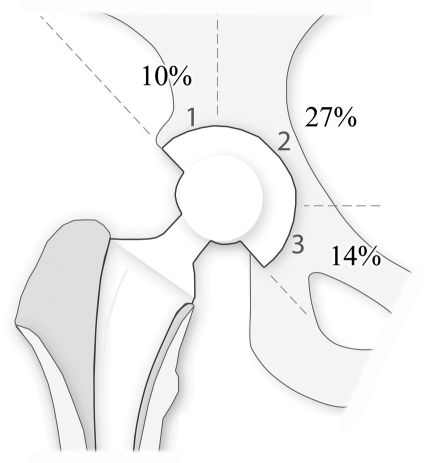
Radiolucencies around the acetabulum in the RingLoc group.

In the 3 zones around the acetabular component, progressive radiolucent lines were seen in 25% of the cups. Most radiolucent lines were located in zone 2 (Figures 2 and 3), with group H showing more lines in zone 2 than group R (43% vs. 27%). 3 cups (3%), 1 in group H and 2 in group R, showed progressive radiolucent lines of more than 3 mm in all 3 zones, indicating a radiographically loosened cup.

### Clinical results

The mean preoperative Harris hip score was 43 (SD 15) and the mean postoperative score at final follow-up was 83 (SD 19).

## Discussion

The newer, second-generation Mallory Head acetabular cup did not have a higher mean survival time than the first-generation cups in patients younger than 55 years. Also, we did not find any improvement in linear wear rates, number of radiographic failures, or clinical outcome.

Our survival analysis suggests that the RingLoc system is not an improvement over the HexLoc system. No statistically significant difference in mean survival time between the groups could be found. The log rank test indicated that there were no differences between the 2 groups, and the difference in risk was close to 0 (0.5%) with a fairly narrow confidence interval (–13 to 14%), indicating that no clinically significant improvement had been made (Nelissen et al. 1992).

A possible explanation for the poor reported survival of the HexLoc system is the cylindrical shape of the acetabular cup, which causes the liner to be asymmetric and thinner at the edges (Hozack et al. 1996). However, the newly designed acetabular cup with the RingLoc system does not have this liner incongruence. No obvious design flaws are apparent in the RingLoc design.

Comparison of cementless acetabular component survival in the literature shows good mean survival rates at 5 years, with declining values after this medium-term result Eskelinen et al. (2006). The same is true of both acetabular components studied here. Unfortunately, only a few cementless cups have well-documented 10-year survival. Eskelinen et al. (2006) reported results of different cementless cups in young patients with the survival at 10 years varying between 71% and 87%. The mean survival times of 89% and 92% at 10 years reported here suggest that this design does perform reasonably well in our cohort of young patients—as compared to other designs—at 10 years. Only 1 report, from the Finnish Arthroplasty Register (Eskelinen et al. 2006), gave 10-year survival of the Mallory Head cup with a HexLoc system. The authors reported a 10-year survival of 61% with revision for any reason as the endpoint. Our reported 10-year survival of the HexLoc system of 89% is substantially higher. One explanation could be that the distribution of preoperative diagnoses (Helse-Bergen HF, 2007)—with a relatively large group of patients with multiple lower extremity joint involvement in our study (e.g. rheumatoid arthritis)—can lead to a less active group of patients. This less active group would be more likely to have less liner wear, and subsequently less prosthetic loosening and lower revision rates. Our subanalysis showed a slightly lower annual liner wear rate (0.12 mm/year vs. 0.17 mm/year) for patients with rheumatoid arthritis, which supports this hypothesis. The higher prevalence of rheumatoid arthritis patients was due to tertiary referral to our university medical center.

The annual liner wear reported in the literature ranges from 0.11 mm/year (Yamamoto et al. 2003) to 0.14 mm/year (Puolakka et al. 2001) for 28-mm heads. This is slightly less than in our group, but unexpectedly, this did not result in a lower mean survival at 10 years. Comparing the 2 generations of acetabular components, the wear analysis showed an equal amount of wear in both groups, suggesting that the increased thickness of the liner at the acetabular metal shell edge had no effect on the wear rate measured. Thus, thinning at the edge of the polyethylene liner, due to the cup-liner incongruence in the HexLoc design, may not be an important issue.

No fair comparison of radiographic changes, except annual wear rate, between the 2 generations of acetabular component could be made at the last follow-up. This is due to the fact that the H group had a longer mean follow-up time, and thus a longer exposure time to wear, than the R group.

A possible source of bias in this study was that both groups included patients who had had previous hip resurfacing arthroplasty. The reason that these patients were included is that they had only had cementless resurfacing hip surgery and not a conventional total hip arthroplasty. Although revision surgery in general suggests a higher re-revison rate, in this “revision” group, none of the metal-backed acetabular components had to be revised and only 6 liner revisions had to be performed. The 10-year survival for these patients was even slightly better than for those with no previous resurfacing (91% vs. 90%), but this was not statistically significant. None of these metal-backed components had to be revised, and the 10-year survival was comparable, which suggests that in our study these hip resurfacings did not have any negative influence on the results.

Our findings confirm that the Mallory Head porous coated femoral component gives excellent results in young patients. Furthermore, in contrast to the alarming reports in the literature (Hozack et al. 1996, Puolakka et al. 1999, 2001), our results suggest that the Mallory Head cementless acetabular components are a reasonable choice for young patients. They also indicate that the RingLoc system does not provide any clinically relevant improvement over the moderate results of the older Hexloc system, despite the correction of the potential design flaws.
